# Workplace- and Residence-Associated Leptospirosis: A Case Report and Review of Current Literature

**DOI:** 10.7759/cureus.29879

**Published:** 2022-10-03

**Authors:** Lilia Shaban, Bahtiyar Toz, Adesh Ramdass

**Affiliations:** 1 Internal Medicine, Icahn School of Medicine at Mount Sinai, Queens Hospital Center, New York, USA

**Keywords:** acute kidney failure, enzyme linked immunosorbent assay (elisa), medical icu, tropical infectious diseases, timing of dialysis initiation, leptospirosis, weil's disease

## Abstract

Leptospirosis is a zoonotic infection most commonly occurring in tropical regions through contact with water or soil contaminated with animal urine. In New York, approximately one to three cases occur annually, the majority occurring through workplace exposure to animal reservoirs. In cases of the more severe presentation of leptospirosis, Weil’s disease, it is important to identify the infection promptly to allow for early antibiotic initiation as well as early initiation of daily dialysis in cases in which it is necessary. We present a case of Weil’s disease in a 64-year-old male with presumed exposure through his combined workplace and residential environment. The resolution of symptoms occurred through a combination of hemodialysis, doxycycline, and meropenem antibiotic treatment. We also discuss the barriers to diagnosis, including the non-specific presentation of leptospirosis, the limitation of access to testing centers, and the limitations to antibody testing within the first week of symptom presentation due to low antibody levels.

## Introduction

Leptospirosis is an infection with the zoonotic spirochete *Leptospira* that can be transmitted most commonly through water or soil contaminated with urine from reservoirs such as farm animals or rodents [1]. Leptospirosis typically presents in tropical or temperate climates, and in the United States, approximately 100-200 cases are reported annually, with the highest incidence occurring in Hawaii [2]. The New York State Department of Health reports about one to three cases of leptospirosis per year, most commonly transmitted to those with close contact with rat urine, often workers in the sewage, agricultural, or abattoir fields, the unhoused populations, as well as those with a history of travel to tropical environments [3]. In 2017, the first and only non-sporadic cluster of infections in New York occurred in a residential building with harborage conditions, defined as conditions in which pests, insects, and rodents can feed, shelter, and reproduce [4]. There is an increased risk of infection in disadvantaged inner-city environments where rodent infestations are more likely, such as the building in which the cluster of infections occurred [5]. 

With respect to transmission, the bacteria can be acquired through open wounds, mucous membranes, or infected water inhalation. It will be transported in the body first through lymphatic circulation before the hematogenous spread of infection to the liver or kidneys [6]. The infection can present in anicteric or icteric forms. The anicteric form, which accounts for 90% of infections, is a less severe form of the disease that will present with non-specific flu-like symptoms and will most likely self-resolve. Common symptoms include fever, headache, myalgia, conjunctival suffusion, abdominal pain, vomiting, diarrhea, and rash. The icteric form is the more severe infection, also referred to as Weil’s disease, and can present with renal and liver failure, hemorrhage, respiratory distress, jaundice, and disseminated intravascular coagulation [7]. 

Diagnosis can be made by isolation in blood or urine cultures, serology, and molecular tests [7-8]. However, treatment should be initiated once there is a suspicion of infection. In addition to symptomatic treatment, oral antibiotics such as doxycycline, amoxicillin, or ampicillin are routinely used. In severe cases, IV penicillin G, third-generation cephalosporins, or erythromycin may be needed [9]. Regional vaccines in France and China have been developed against leptospirosis, but limitations based on serovar differences, and vaccine safety, efficacy, and cost are still of concern [5].

## Case presentation

A 64-year-old male with a past medical history of hypertension, hyperlipidemia, and alcohol use disorder presented for complaints of a five-day history of generalized weakness, dyspnea, lightheadedness, cough productive of white sputum, decreased urine output, arthralgias, non-bilious, non-bloody vomit (20 episodes in two days), generalized abdominal pain, and bilateral lower extremity pain. He denied any hemoptysis, diarrhea, constipation, rash, orthopnea, or loss of consciousness. The patient works as a building superintendent taking out the trash and performing repairs. In addition, he lives in the basement of the site where he works. He has a smoking history of 14 pack-years and quit 12 years prior. The physical examination noted bilateral pulmonary basal crackles, tachycardia, scleral icterus, right upper quadrant tenderness, and dry mucous membranes. Physical examination revealed a fever of 103.4 °F, a heart rate of 143, and blood pressure of 88/67, and his mental status began to deteriorate as he became increasingly confused. Laboratory results revealed leukocytosis with a white blood cell (WBC) count of 16.59 x 10^3^/ mcL, thrombocytopenia (42 x 10^3^/ mcL), increased blood urea nitrogen level (BUN) of 81 mg/dL, creatinine of 5.59 mg/dL, D-dimer level of 456 ng/mL, pro B-type natriuretic peptide (BNP) of 23 701 pg/mL, and a decreased estimated glomerular filtration fraction (eGFR) of 10 mL/min/1.73m2. A hepatic function panel also revealed signs of liver function deterioration with a decreased albumin level of 2.3 g/dL, increased alanine aminotransferase level (ALT) of 63 U/L, and an increased aspartate transaminase level (AST) of 132 U/L. The patient was then transferred to the intensive care unit (ICU) under suspicion of sepsis and acute kidney injury of unknown origin. The patient was initiated on IV azithromycin (500 mg), IV ceftriaxone (1000 mg), and IV norepinephrine (2-25 mcg/min). He also received an initial 4 liters of IV fluid without return to hemodynamic stability. A high-flow nasal cannula was required to maintain adequate oxygenation, and imaging revealed unremarkable findings of the kidneys on non-contrast CT scans of the abdomen and pelvis. 

The patient then began to exhibit bleeding of the gums in a pinpoint pattern. Heparin was originally initiated due to suspicion of pulmonary embolism but at this stage of management, it was discontinued as the platelet count further decreased to 32 x 10^3^/mcL. The patient was transfused one unit of platelets with a subsequent rise in platelet count to 48 x 10^3^/mcL. Leukocytosis continued to worsen as the WBC count reaches 25.99 x 10^3^/mcL, and renal function continued to deteriorate with a BUN of 149 mg/dL and a creatinine level of 8.48 mg/dL. Due to deterioration of renal function, the patient was initiated on daily hemodialysis, which continued for five days before adjusting to a schedule of every other day for a total of seven sessions during the hospital course. A further history obtained from the patient’s girlfriend revealed a concern for infection due to the possibility of "eating something with mice urine” due to his living environment. A suspicion of leptospirosis was raised after the history and worsening renal and liver conditions. 

IV doxycycline (200 mg) and IV meropenem (500mg) were initiated empirically for a 10-day course based on suspicion of leptospirosis while testing for leptospirosis antibodies was conducted. Leptospirosis IgM results returned as positive; the 10-day course of antibiotics was completed with improved respiratory, renal, and hepatic function, including resolution of icterus, thrombocytopenia, and improved urine output.

## Discussion

The diagnosis of leptospirosis can be challenging to identify because laboratory indications are non-specific, and the disease is uncommon in non-tropical regions. Patients may present with elevated erythrocyte sedimentation rates, thrombocytopenia, leukocytosis, hyperbilirubinemia, elevated creatinine, and elevated amylase [10]. These abnormalities can allow for the confusion of leptospirosis with more common illnesses, especially in the non-severe form, which can present like the flu. In the severe form, it may resemble illnesses from tick-borne infections, for example, *Anaplasmosis* which can present with pulmonary manifestations, thrombocytopenia, fever, and liver enzyme derangements [11]. This highlights the importance of obtaining a thorough patient history for risk factors and potential exposure. 

The patient's clinical presentation demonstrated Weil’s disease, a more severe form of leptospirosis that may present with acute hepatic failure, acute kidney injury, acute respiratory distress syndrome, and diffuse alveolar hemorrhages [10]. The pathogenesis of the disease is thought to be due to insult to the endothelial lining of small blood vessels, which can explain manifestations such as interstitial nephritis, tubular/glomerular/vascular renal pathologies, and injury to hepatic capillaries [10]. Patients with the more severe Weil’s disease admitted to the ICU most often require hemodialysis to compensate for the uremia induced by acute kidney injury, as demonstrated by the presented patient. There is evidence that patients who receive a prompt “door to dialysis” period from ICU exhibit a significant difference in mortality rates and decreased duration of ICU stay compared to those that received delayed alternate day dialysis. This demonstrates that maintaining strict control of azotemia and fluid volume is associated with improved survival rates [12]. 

Difficulties with the diagnosis can occur due to limited resources and testing centers. Leptospirosis can be diagnosed by enzyme-linked immunosorbent assay (ELISA) or microscopic agglutination tests (MAT) for antibodies. However, rapid tests such as an IgM “dot blot” can be used, such as in the case of the presented patient. Difficulties can arise due to a lack of antibodies in the acute phase of the disease, which is why in recent years, polymerase chain reaction (PCR) assays have been used to detect antibody levels in the early phase of infection [13]. The MAT method has a sensitivity of 41% within the first week, 82% during the second to fourth week, and 96% beyond the fourth week of illness. The MAT method depends on the different serovars, whereas the ELISA may be positive six to eight days before the MAT results. With rapid screening tests such as the dot blot, positive results may not be detectable until the second week after symptom onset, as they have low sensitivity in the acute phase of infection [13]. The presented patient had collections for a dot blot test collected three days after admission to the hospital, and the patient identified the onset of symptoms five to seven days before admission to the hospital, placing the blot test within a presumed second week of infection. Barriers to detection are even more prevalent in developing countries in which the prevalence of leptospirosis may be higher, but access to testing centers is decreased [13]. This illustrates a need for more accessible and time-sensitive forms of diagnostic detection to combat the limited laboratories and costs related to the maintenance of Leptospira serovars [5]. 

Access to more widely available and convenient diagnostic methods can aid in early detection, especially in high-risk environments and occupations. This can lead to improved patient outcomes with the quicker onset of treatment initiation, which has been shown to reduce the duration and severity of illness [5].

## Conclusions

Leptospirosis can present with many difficulties and barriers to diagnosis, including limited availability of testing centers and non-specific disease presentation leading to delayed treatment. It is important to target efficient and easily accessible diagnostic methods so that prompt antibiotic initiation or treatment such as dialysis can be administered, especially in the case of Weil’s disease. A shorter door-to-dialysis period in the setting of Weil’s disease offers an improved patient recovery time and outcome.
